# Dielectric Response of Micelles Built from Intrinsically Disordered Beta-Casein

**DOI:** 10.3390/ijms27198810

**Published:** 2026-10-01

**Authors:** Yuriy F. Zuev, Denis A. Smolei, Polina V. Skvortsova, Liliya R. Bogdanova, Artur A. Galiullin, Olga S. Zueva, Svetlana R. Derkach, Ivan V. Lunev

**Affiliations:** 1 Kazan Institute of Biochemistry and Biophysics, FRC Kazan Scientific Center, Russian Academy of Sciences, Lobachevsky Str. 2/31, Kazan 420111, Russia; denissmolej@gmail.com (D.A.S.); skvpolina@gmail.com (P.V.S.); chemli@mail.ru (L.R.B.); 2 Institute of Physics, Kazan (Volga Region) Federal University, Kazan 420021, Russia; www.ag95@mail.ru (A.A.G.); lounev75@mail.ru (I.V.L.); 3 Institute of Electric Power Engineering and Electronics, Kazan State Power Engineering University, Krasnoselskaya Street 51, Kazan 420066, Russia; ostefzueva@mail.ru; 4 Institute of Natural Sciences and Technology, Murmansk Arctic University, Sportivnaya Str. 13, Murmansk 183010, Russia; derkachsr@mauniver.ru

**Keywords:** disordered proteins, dielectric relaxation, beta-casein, micelles, free and bound water

## Abstract

For many decades, protein function was associated with a rigid three-dimensional structure. However, in recent years, interest has grown rapidly in proteins that successfully perform their functions despite being intrinsically disordered. Unfortunately, to date, insufficient structural information has been collected on this novel protein family to analyze its structure–function correlations. To obtain the required structural information on intrinsically disordered beta-casein (b-CN), we used broadband dielectric spectroscopy, which has long been used to study structured proteins. Dielectric measurements in the frequency range from 1 Hz to 67 GHz detected three Cole–Cole relaxation processes in a micellar solution of disordered beta-casein. The experimental data obtained were used to estimate the dynamical hierarchy of dipole–dipole correlations involved in the relaxation of each dipole component of the system. The dielectric relaxation of micellized disordered b-CN is rather complex, indicating the cascade nature of protein relaxation, which involves long-range structural correlations of flexible charged protein chains. The relaxation of free and bound water is close to the Debye type, which is entirely consistent with the “wait and switch” model of dielectric relaxation, in which a water dipole waits for the arrival of a structural or ionic defect for reorientation. The relaxation rates of the two water fractions differ dramatically, with bound-water relaxation being two orders of magnitude slower than free-water relaxation. The most reasonable explanation for this difference probably lies in the spatial confinement of bound water resulting from the influence of the charged protein surface. The results obtained demonstrate the research potential of broadband dielectric spectroscopy not only for long-studied rigid, structured proteins with permanent dipole moments but also for the novel family of disordered proteins with fluctuating dipole moments.

## 1. Introduction

Since their discovery more than a quarter of a century ago [1], interest has grown rapidly in proteins that successfully perform their functions while being intrinsically disordered, i.e., lacking a fixed or ordered three-dimensional structure [2,3]. Unfortunately, to date, insufficient structural information has been collected on this new protein family to analyze its structure–function correlations. Intrinsically disordered proteins (IDPs) are found in different protein families, including milk proteins, one of which is the subject of the present study [4]. Caseins account for a considerable proportion of the total protein in bovine and human milk [5,6]. Caseins are present and function in milk in the form of colloidal particles composed of associated proteins and calcium phosphate, known as casein micelles [7], in which the intrinsically disordered nature of caseins supports their physiological functions [8].

Water plays an integral role in the colloidal and functional properties of caseins. Hydration determines the dynamic structure and stability of biological macromolecules and their functional supramolecular complexes [9,10]. The surface hydration of biopolymers governs their structural stability and flexibility, while correlated water-macromolecule fluctuations are essential for biological function. In biological systems, numerous solutes and confinement cause water molecules to acquire specific physical properties [11,12], including changes in rotational and translational mobility. Beyond their functional significance, water–biopolymer interactions also provide insights into the use of water’s altered physical properties as biomarkers of the structural state across different biological systems [13,14].

Despite numerous experimental studies of the intrinsically disordered nature of different caseins, many details of their supramolecular colloidal properties remain unclear. The subject of the present study is the correlated dynamics of water and β-casein (b-CN) in a micellar solution of this milk protein. b-CN is a member of the milk casein family, together with α_s1_-, α_s2_-, and κ-caseins. All four caseins, together with calcium phosphate, form casein micelles, whose main physiological function is to transport essential minerals, amino acids, and fats and supply them to mammals.

**Figure 1:**
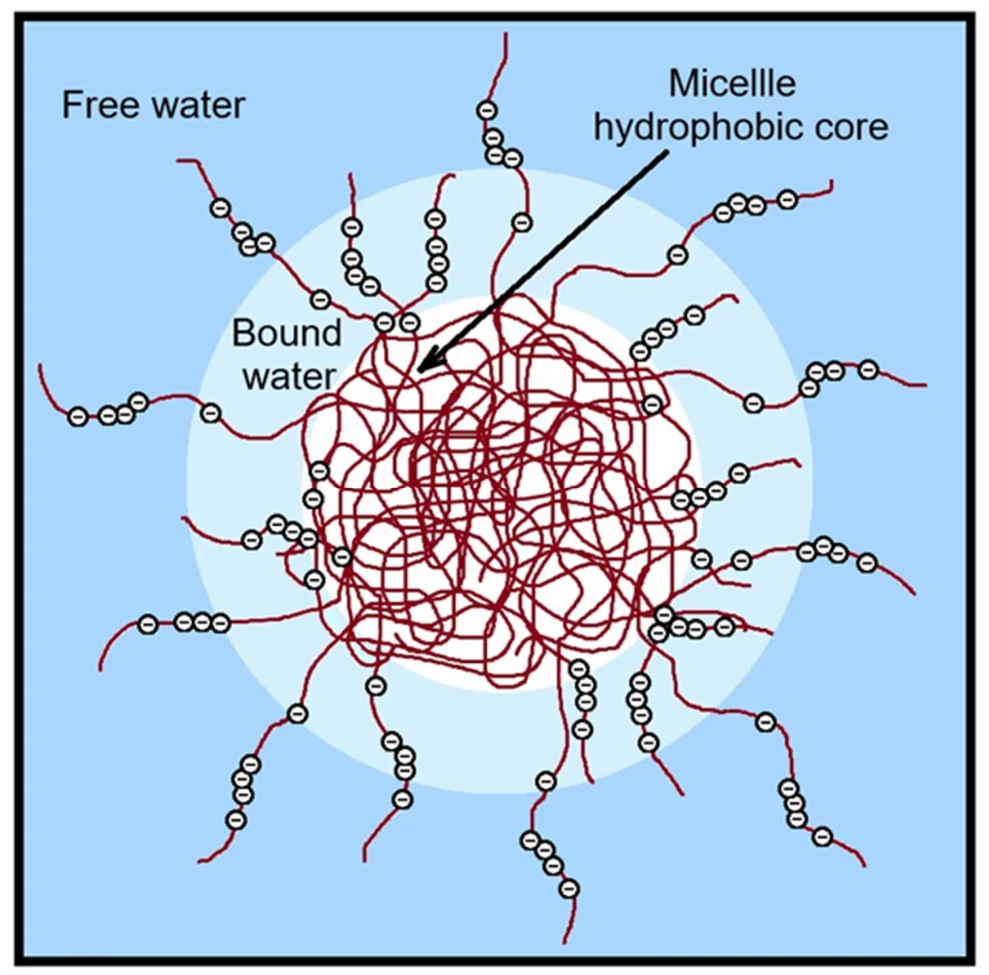
Schematic representation of a b-CN micelle. The white sphere shows the hydrophobic core of the micelle, formed by nonpolar amino acid residues. The light blue region represents the hydrophilic surface layer of the micelle, formed by polar amino acids, including negatively charged phosphoserines.

The structure of multicomponent casein micelles is rather complex. For this reason, individual types of caseins are often used to study different aspects of casein micellization [15,16,17]. The b-CN molecule is a linear, single-stranded polypeptide [18] that is classified as an intrinsically disordered protein (IDP) [19]. Most of the protein’s electric charges are concentrated in its polar N-terminal region, whereas the C-terminal region consists predominantly of nonpolar hydrophobic amino acids. Owing to its amphiphilic molecular structure, b-CN behaves as a natural surfactant and self-associates into micelle-like aggregates, similarly to an ordinary ionic surfactant [20,21,22,23,24]. The nonpolar fragments of b-CN molecules form the hydrophobic core of the micelle, while their polar fragments interact electrostatically with each other, stabilizing the shape of the micelle in the aqueous environment (Figure 1).

**Figure 2:**
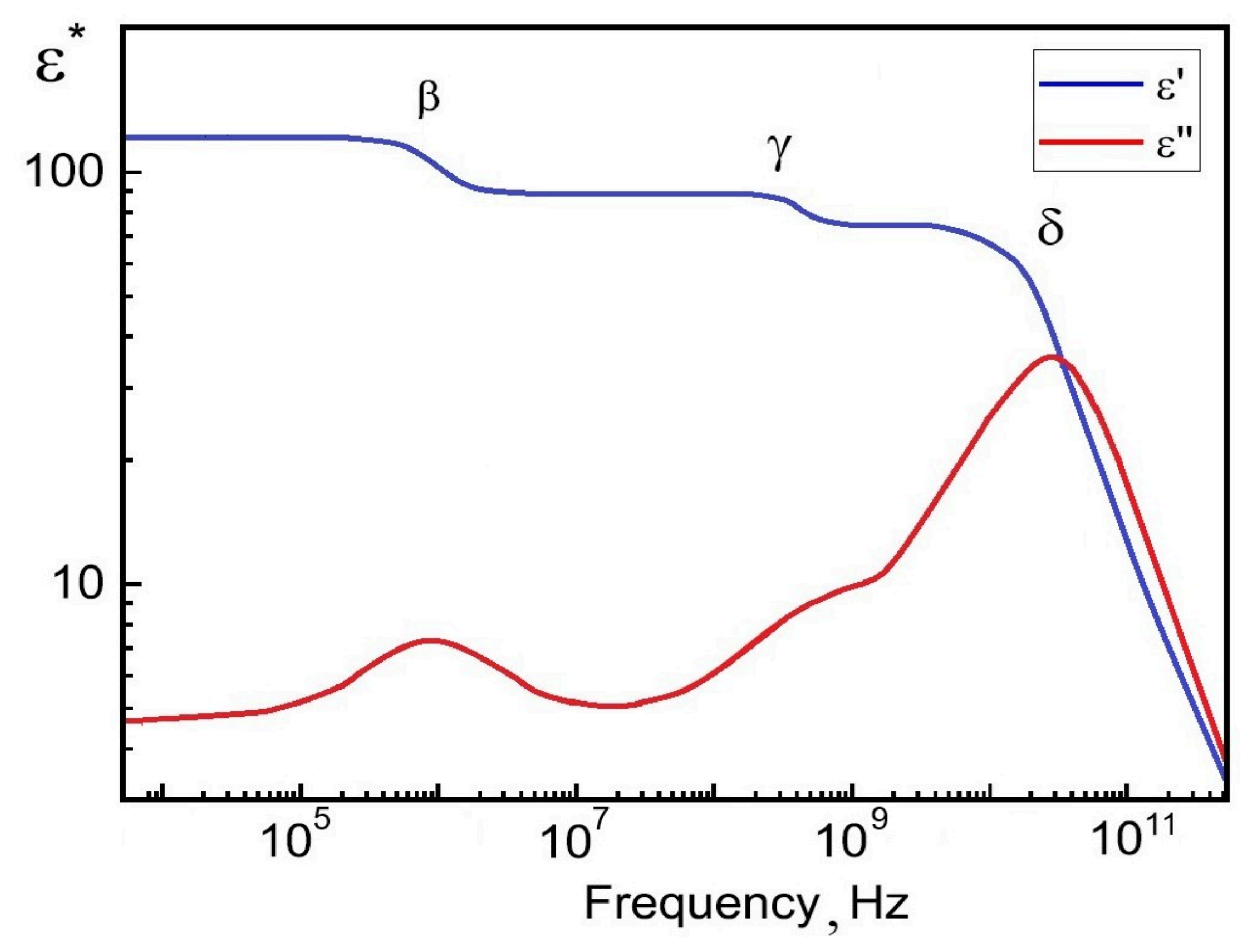
Schematic representation of the dielectric spectrum of an aqueous protein solution with β-, γ-, and δ-dispersions located in the frequency range 10^4^–10^12^ Hz. Dielectric permittivity is shown in blue and dielectric loss in red.

To investigate the dynamic structure of hydrated b-CN micelles, broadband dielectric spectroscopy (BDS) was used in this study. Extensive experience with BDS studies of the dielectric properties of protein solutions has shown that these systems exhibit three main relaxation processes, schematically represented in Figure 2. The β-dispersion is due to protein relaxation, the δ-dispersion reflects the relaxation of water molecules bound to the protein, and the γ-dispersion arises from the relaxation of free water [25,26,27]. The analysis of dielectric data for any biological system is not trivial. Indeed, separating and reliably assigning individual relaxation contributions in multicomponent systems is challenging because of their complex composition, the diversity of relaxation mechanisms, and the nonselective, broadband nature of dielectric relaxation. For example, a protein can be a rigid globular molecule with a permanent dipole moment. In this case, the dielectric relaxation of the protein is usually caused by molecular tumbling controlled by hydrodynamic friction with the solvent [26,27,28]. Another case involves proteins that completely or partially lack a rigid structure, a feature that gives the protein backbone flexibility and plasticity [29]. To date, we are unaware of experimental studies of dielectric relaxation processes in disordered proteins. However, a highly relevant computational study was recently conducted, modeling the dielectric response of IDPs by combining molecular dynamics simulations with formal theories [30]. It was shown that IDPs can produce large fluctuating dipole moments owing to the conformational flexibility of the protein chain, thereby making a significant contribution to the dielectric permittivity of the protein solution in the form of β-dispersion.

The dielectric properties of protein hydration water are also considerably diverse. In most cases, the bound or hydration water, which differs in its relaxation properties from bulk free water, is grouped into a single category [31,32,33]. In other cases, the complexity of the dispersion curves is explained by dividing hydration water into loosely and tightly bound fractions with different degrees of protein binding and, consequently, different mobilities [34,35,36].

The main objective of this work was to experimentally study the disordered protein over a wide range of electromagnetic frequencies to obtain comprehensive information on the dynamical structure of the protein and its aqueous environment. We sought to address two main objectives: to obtain new data on the dynamical molecular structure of the disordered milk protein beta-casein and to assess the ability of dielectric spectroscopy to study polarization associated with the protein’s fluctuating dipole moment.

## 2. Results and Discussion

In general, the dielectric spectrum $\varepsilon *\left(\omega \right)$ of any complex aqueous system can be described by a sum of *j* dielectric relaxation processes [37]:

(1) $$\varepsilon *\left(\omega \right)\;=\;\varepsilon^{\prime }\left(\omega \right)\;–\;i\varepsilon^{\prime \prime }\left(\omega \right)\;=\;\varepsilon_{\infty }+\;\sum_{j}\frac{\Delta \varepsilon_{j}}{{\left\{1\;+\;{\left(i\omega \tau_{j}\right)}^{\left(1-\alpha_{j}\right)}\right\}}^{\beta_{j}}}$$

where $\varepsilon^{\prime }$ and $\varepsilon^{\prime \prime }$ are the real and imaginary parts of the complex permittivity ε*, ω is the cyclic frequency of the applied electromagnetic field, *i* is the imaginary unit, $\varepsilon_{\infty }$ is the high-frequency limit of the complex dielectric permittivity, $\Delta \varepsilon_{j}=\varepsilon_{sj}-\varepsilon_{\infty j}$ is the amplitude (dielectric strength), $\tau_{j}$ is the relaxation time, α*_j_* and β*_j_* are the band-shape parameters of the dielectric process [38]. When α = 0 and β = 1, the relaxation process in Equation (1) is of the Debye type [39]. For aqueous protein solutions, three main dielectric relaxation contributions are usually detected in the kilohertz to gigahertz frequency range (Figure 2) with symmetrical broadening of the relaxation peak, corresponding to the Cole–Cole equation with 0 ≤ α < 1 and β = 1 [40], which, for simplicity, is sometimes written as [41]

(2) $$\varepsilon *\left(\omega \right)=\varepsilon_{\infty }+\frac{\varepsilon_{s}-\varepsilon_{\infty }}{1+{\left(i\omega \tau \right)}^{\left(1-\alpha \right)}},$$

where $\varepsilon_{s}$ is the static dielectric permittivity. The case α = 0 corresponds to Debye-type relaxation. The relaxation time τ and the Cole–Cole parameter α qualitatively describe the dynamics of the relaxation process. By contrast, the dielectric strength Δε is a measure of the dipolar structure of the studied system, as described by the Kirkwood–Fröhlich equation [42]:

(3) $$\frac{\left(\varepsilon_{s}-\varepsilon_{\infty })(2\varepsilon_{s}+\varepsilon_{\infty }\right)}{\varepsilon_{s}{\left(\varepsilon_{\infty }+2\right)}^{2}}=\frac{N}{3\varepsilon_{0}}\frac{\langle {\vec{M}}^{2}\rangle }{3k_{B}T}$$

where $\varepsilon_{0}$ = 8.85 × 10^−12^ F/m is the dielectric constant, $k_{B}$ is the Boltzmann constant, *T* is the absolute temperature, *N* is the volume concentration of relaxing dipoles, $\vec{M}=\sum_{i=1}^{N}g{\vec{m}}_{i}$ is the effective dipole moment of a unit volume, g = 1 + z〈cos θ〉 is the Kirkwood correlation factor with z and θ denoting the number of nearest interacting dipole neighbors and the average angle between their dipole moments, respectively [42]. In a multicomponent dipolar system, this expression can be applied to every detected relaxation process.

**Figure 3:**
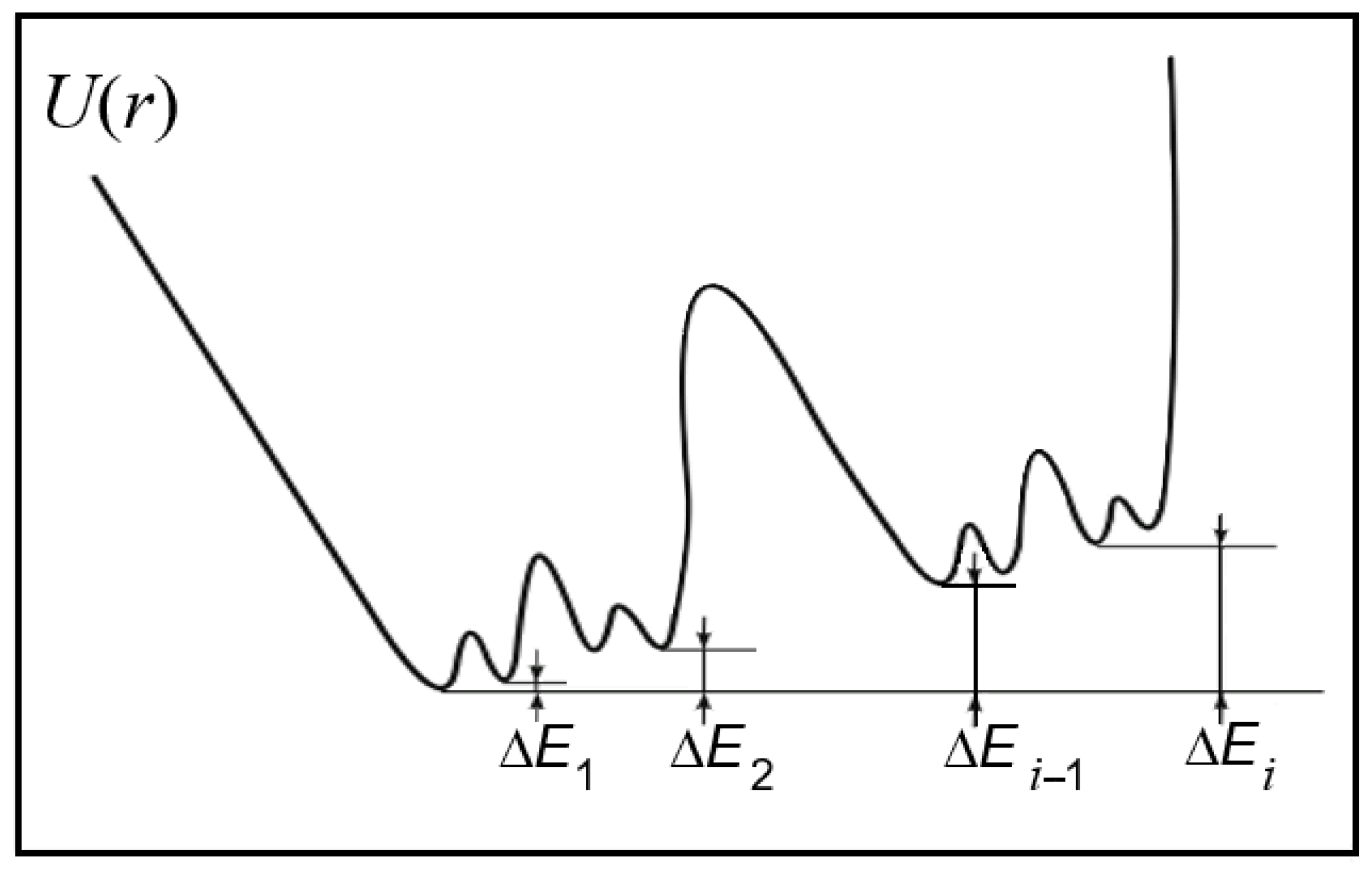
The potential barrier profile *U(r)* of dipole relaxation in an external electric field [48].

Previously, a modified approach for analyzing dielectric relaxation in protein solutions was proposed, based on the broadening of relaxation peaks resulting from the structural composition of a dipolar system [43,44,45,46,47]. According to the proposed model, the Cole–Cole broadening parameter α has a clear physical interpretation as a signature of dipole–dipole correlation, expressed as the fractal dimension of the dipole reorientation process [45,46]:

(4) $$\alpha =\frac{\ln N_{\tau }}{\ln(\tau /\tau_{0})}$$

where τ is the experimental relaxation time of the dipole ensemble, $\tau_{0}$ is the reorientation time of a single dipole, $N_{\tau }$ is the number of reorientation events required for complete relaxation in the observed relaxation process, or the relative spatiotemporal correlation of dipole–dipole interactions among the relaxing dipoles. In other words, in this model, the Cole–Cole parameter is interpreted as a fractal dimension that describes the hierarchy of elementary relaxation events sufficient to achieve complete relaxation of the examined dipole component [48,49]. The relaxation kinetics in such a model follow the hierarchy of relaxation times for the rearrangement of correlated particles (elementary dipoles), which is schematically shown in Figure 3. In the case of $N_{\tau }$ = 1, α = 0, Debye relaxation occurs, and all individual dipoles relax independently. By contrast, when dipole reorientation depends on correlations with surrounding dipoles, the parameter satisfies 0 ≤ α < 1, and for complete relaxation of the examined dipole fraction, several successive rearrangements must occur, which are represented by the number of elementary reorientation events $N_{\tau }$. As a result, this mechanism prolongs the relaxation process and manifests experimentally as broadening of the relaxation peak.

The subsequent analysis of the experimental results involves two main calculations. The first is based on the Fröhlich function *B*(*T*) [44,50]:

(5) $$B(T)=\Delta \varepsilon (T)\frac{2\varepsilon_{s}(T)+\varepsilon_{\infty }}{3\varepsilon_{s}(T)}=\frac{1}{3\varepsilon_{0}k_{B}V}\langle {\vec{M}}^{2}\rangle$$

where *V* is the unit volume, and the total dipole moment $\vec{M}=\sum_{i=1}^{N}g\mu_{\mathrm{mic}}^{i}$ is obtained by summing the dipole moments $\mu_{\mathrm{mic}}^{i}$ of b-CN micelles within this volume. The equation was used to estimate the dipole moment of a b-CN micelle based on the known protein concentration and the assumed aggregation number of b-CN molecules per micelle. In our calculations of the dipole moment of b-CN micelles, we assumed a spherical shape and a mean cosine of the angle between charged b-CN fragments close to zero (Figure 1), resulting in the Kirkwood correlation factor *g* = 1. The dielectric strength of free and bound water relaxation in Equation (3) was used to estimate the bound-water fraction based on the known value of the water dipole moment.

In addition, we used the measured temperature dependence of the relaxation times and the broadening of the relaxation peaks to estimate the dynamics of molecular processes in the studied system. This analysis combined the Arrhenius behavior of the relaxation time with an examination of the Cole–Cole broadening of the relaxation peaks to estimate the associated spatiotemporal correlation of dipole–dipole interactions, *N*_τ_ (see details in the Supplementary Materials (Figure S1).

**Figure 4:**
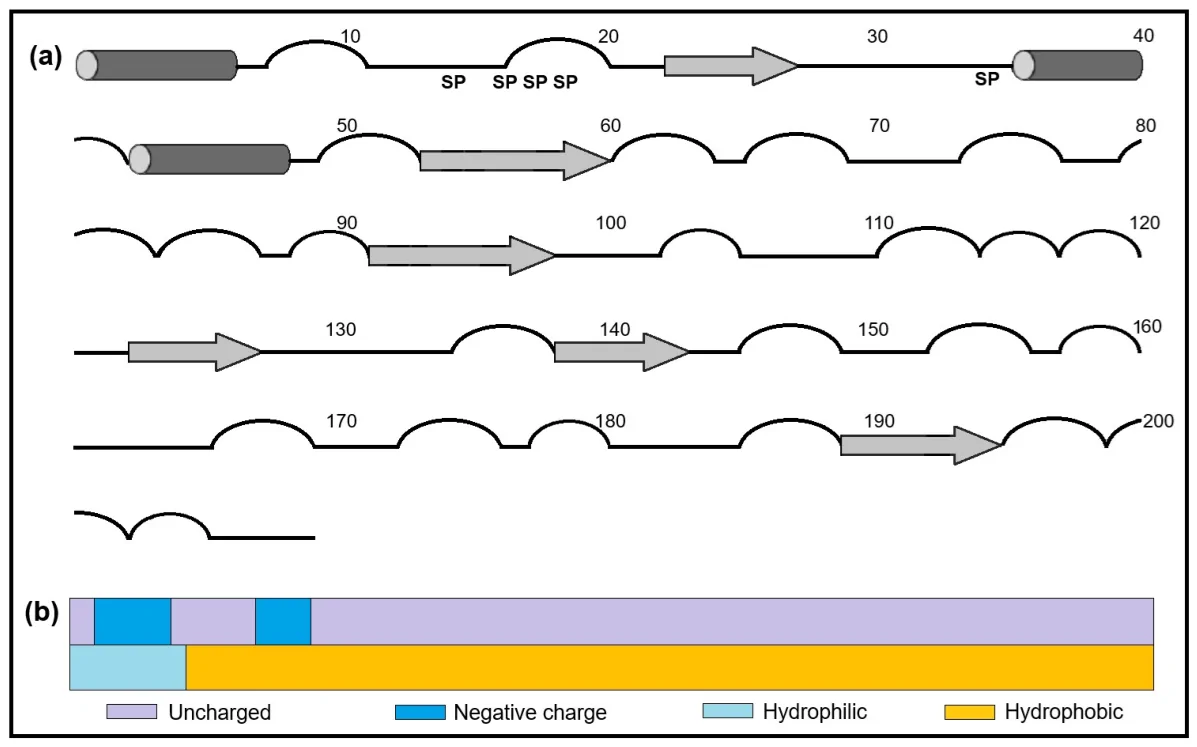
Distribution of b-CN secondary-structure elements along its polypeptide sequence [59] (**a**) and of the charged, hydrophilic, and hydrophobic regions of this linear protein [60] (**b**). SP denotes negatively charged serine phosphate residues; α-helix fragments are shown as cylinders, extended β-strands as arrows, and turns as semi-elliptical elements.

β-Casein (b-CN) is one of the most abundant protein compounds in food products [51,52,53,54,55] and colloidal technologies [15,16,17,18,20,21]. This phosphoprotein, with a molecular mass of about 24 kDa, consists of a polypeptide chain composed of 209 amino acid residues and lacks cysteine residues and intramolecular disulfide bridges (Figure 4a) [56,57,58]. A short N-terminal fragment of b-CN consists mainly of hydrophilic residues carrying nearly all of the protein’s electric charges, whereas the long C-terminal region is mainly hydrophobic and almost electrically neutral (Figure 4b). Because the b-CN molecule contains only a few secondary-structure fragments and completely lacks intramolecular chemical bonds [55,56], the structural amphiphilicity and considerable flexibility of its molecules [22,23,24,59,60] result in their self-association in aqueous solutions and the formation of micelle-like aggregates (Figure 1).

Micellization of b-CN is temperature- and concentration-dependent. For example, the monomer–micelle transition for b-CN used in the present study occurs at 15, 20, and 28 °C for protein concentrations of 1, 0.4, and 0.2 mg/mL, respectively [22]. Thus, the higher the concentration, the lower the transition temperature. The internal structure of b-CN micelles was previously determined using small-angle neutron scattering [20]. According to this study, b-CN micelles consist of a core with a density of 0.4–0.9 g/cm^3^ and an outer corona-like shell with a much lower density of 0.025–0.14 g/cm^3^. The apparent radius of gyration of the micelles was determined to be 13.5 ± 1.0 nm, irrespective of protein concentration and aggregation number. Later, one of the co-authors of the present study confirmed the hydrodynamic radius of b-CN micelles (10 nm) by dynamic light scattering (DLS) [22]. This information was used in the present study as the basis for constructing the schematic representation of a b-CN micelle (Figure 1). In addition to the basic structural model of a b-CN micelle, we included five negatively charged phosphoserines present at the hydrophilic N-terminus of each casein molecule (Figure 4), thereby making the hydrophilic corona-like shell of b-CN micelles highly charged.

**Figure 5:**
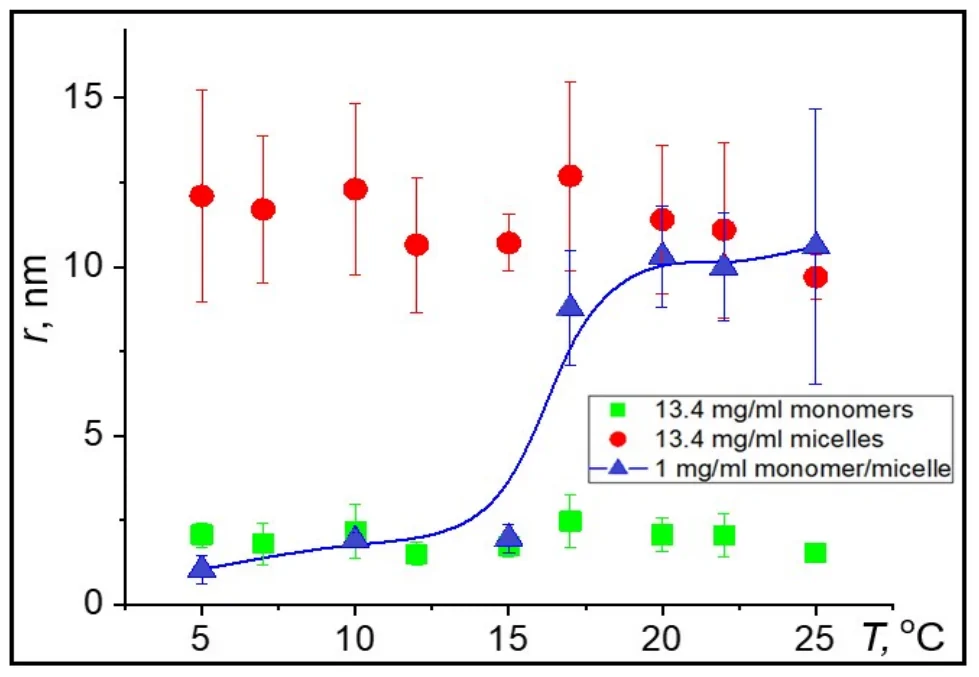
Temperature dependence of the hydrodynamic radius of b-CN molecules and micelles.

We confirmed the micellar state of the studied b-CN system. Figure 5 shows the DLS results for two b-CN solutions with different concentrations. At a low b-CN concentration, we observed the well-known temperature-dependent monomer-to-micelle transition: at approximately 15–17 °C, b-CN molecules self-associated into micelles with a hydrodynamic radius of 10–11 nm. At the higher concentration, the b-CN solution remained in a micellar state at all temperatures, with an average micellar hydrodynamic radius of approximately 11–12 nm. At the same time, monomeric protein was also present, but its relative fraction was less than 10%.

**Figure 6:**
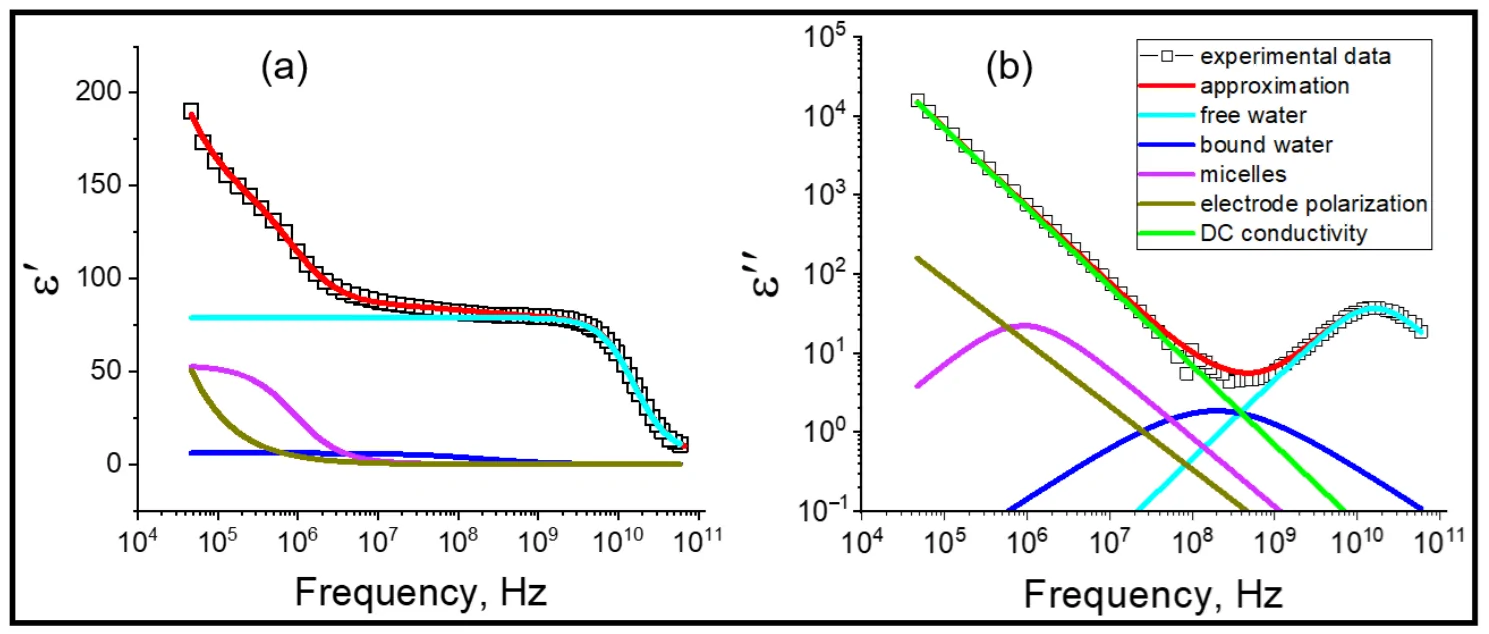
Spectra of dielectric permittivity (**a**) and dielectric loss (**b**) for a 10 mg/mL b-CN solution at 20 °C. The red line represents the fit to the data; the light blue curves represent free-water relaxation; the blue curve represents bound-water relaxation; the purple curve represents b-CN relaxation; the marsh-green curve represents electrode polarization (Jonscher); and the green curve represents DC conductivity.

A typical example of the obtained dielectric results is shown in Figure 6 (additional results are shown in the Supplementary Materials as Figure S2). Both the dielectric permittivity $\varepsilon^{\prime }\left(\omega \right)$ and dielectric loss permittivity $\varepsilon^{\prime \prime }\left(\omega \right)$ spectra were simultaneously approximated using a superposition of three Cole–Cole terms, a conductivity term, and the Jonscher function, which describes the total residual influence of low-frequency processes outside the measurement window, such as electrode polarization [61,62]:

(6) $$\varepsilon *\left(\omega \right)=\varepsilon_{\infty }+\frac{\Delta \varepsilon_{1}}{1+{\left(i{\omega \tau }_{1}\right)}^{\alpha_{1}}}+\frac{\Delta \varepsilon_{2}}{1+{\left(i{\omega \tau }_{2}\right)}^{\alpha_{2}}}+\frac{\Delta \varepsilon_{3}}{1+{\left(i{\omega \tau }_{3}\right)}^{\alpha_{3}}}+A{\left(i\omega \right)}^{n-1}+\frac{\sigma }{i{\omega \varepsilon }_{0}}$$

where subscripts 1, 2, and 3 denote the relaxation processes of b-CN in the micellar state and of bound and free water, σ is the direct current (DC) conductivity, and *A* and *n* are the amplitude and exponent of the Jonscher function, respectively.

**Figure 7:**
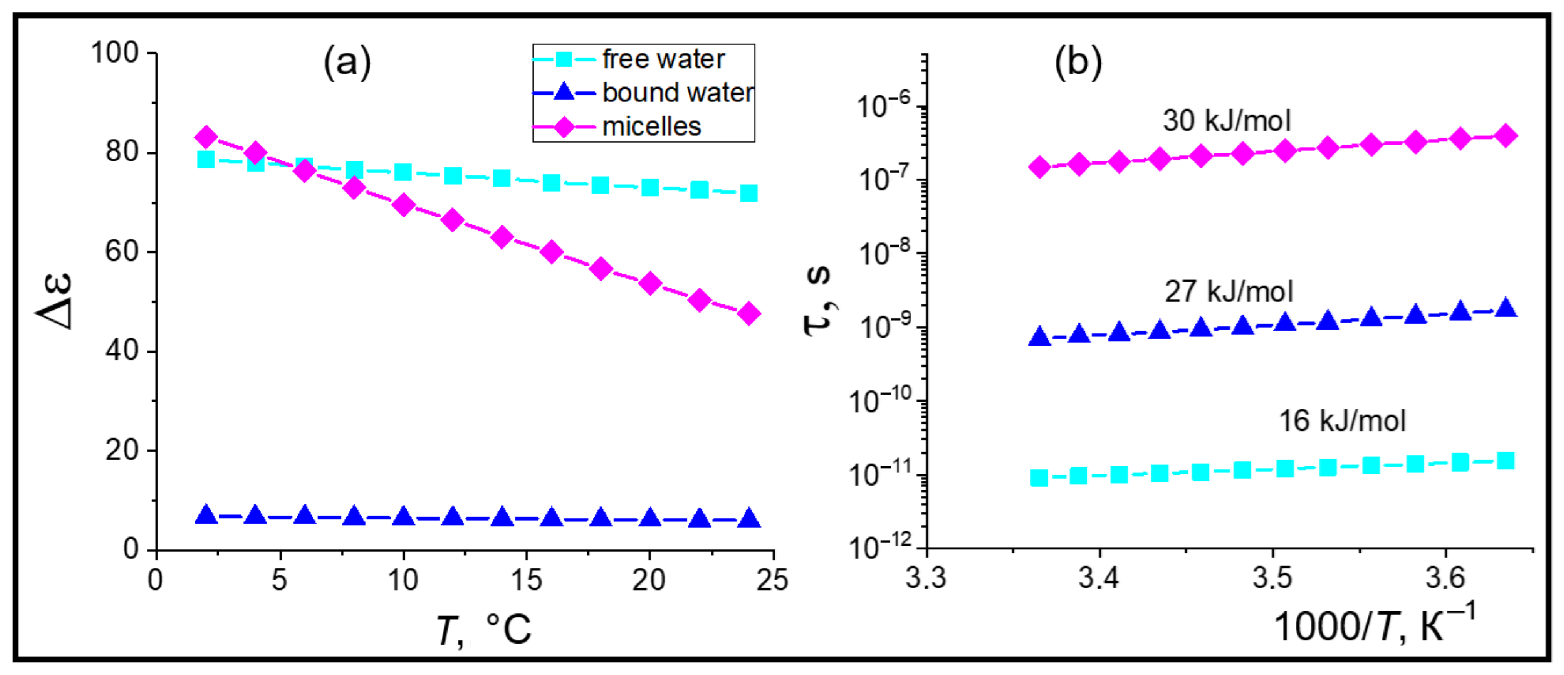
Temperature dependences of the dielectric strength (**a**) and relaxation time (**b**) of the relaxing components in a 10 mg/mL b-CN micellar solution. The errors in determining Δε are 3% for free water, 7% for bound water (the scatter lies within the symbols), and 5% for micelles. The errors for τ are 3–5%.

The relaxation process centered in the microwave-frequency range at tens of GHz represents bulk-water relaxation [63]. Additional evidence supporting this assignment includes the temperature dependence of the dielectric strength [64] and the activation energy of this process, which is 16 kJ·mol^−1^ (Figure 7b), close to the value of 15.9 kJ·mol^−1^ reported for the dielectric relaxation of pure water [65,66].

The lowest-frequency process is assumed to arise from protein relaxation. Typically, dielectric studies have been applied to rigid proteins with a fixed spatial structure and a permanent dipole moment arising from electric charges distributed throughout the protein structure. The dielectric relaxation of structured proteins in aqueous solutions is known to reflect their spatial structure through the protein’s permanent dipole moment, which is proportional to the dielectric amplitude of the process. The relaxation time of protein dipole tumbling (the β-peak in Figure 2) correlates with the hydrodynamic radius of the protein [24,67,68,69]. The β-peak in the dielectric spectrum, whose position is determined mainly by the size of the protein molecule and protein–protein interactions (protein concentration), is often located in the frequency range 10^6^–10^8^ Hz [68,69,70].

Our assignment of the observed low-frequency process to protein relaxation is based on its extremely high dielectric strength (Figure 6a, diamonds), which is atypical of common rigid proteins [67,68,69,70] but characteristic of IDPs [30].

The intermediate relaxation process corresponds to bound, or hydration, water. Most proteins adsorb a certain amount of water on their surface. The relaxation of bound water is observed over a wide frequency range, but it is generally located between the relaxation peaks of the protein and free water. IDPs can have several fractions of bound water that collectively cover a rather broad frequency range, as can be seen in Figure 6 [36,71].

**Figure 8:**
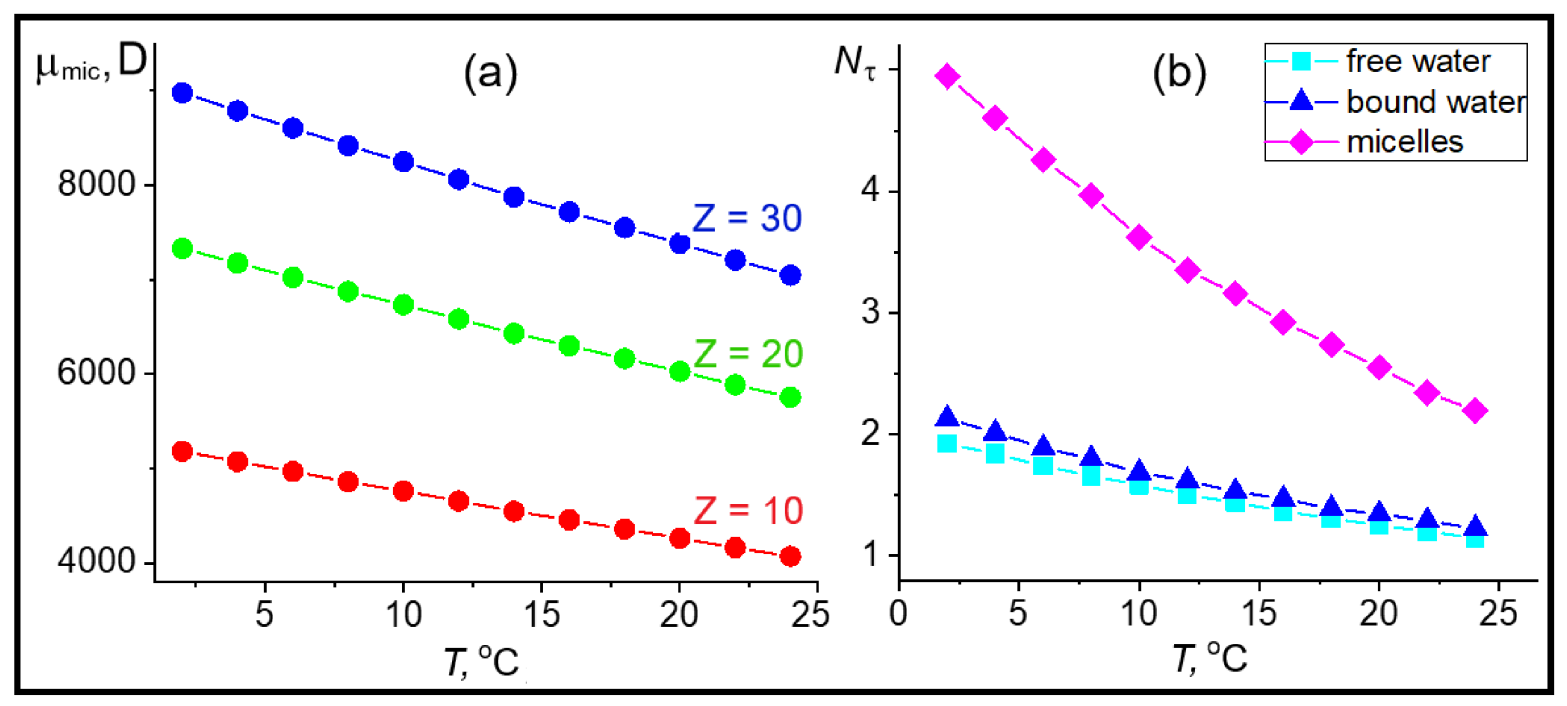
Dipole moment of b-CN micelles at different aggregation numbers Z (**a**); the number of reorientation events required for complete relaxation of free water, bound water, and b-CN micelles, *N*_τ_ (**b**). The b-CN concentration was 10 mg/mL.

The relationship between the dielectric strength of the relaxation process, Δε, and the characteristic dipole moment is well described by the Kirkwood–Fröhlich equation [40,44,72]. Equation (3) was used to calculate the effective dipole moment of b-CN micelles (Equations (S1) and (S2) in Supplementary Materials) and to estimate the amounts of free and bound water in the b-CN micellar solutions, i.e., the degree of micelle hydration. In these calculations, we used the known dipole moment of a water molecule in the gaseous phase, 1.84 D, and its Kirkwood correlation factor, *g* = 2.8 [28,66]. The values of $\Delta \varepsilon_{j}$ and the corresponding high- and low-frequency limits of the complex dielectric permittivity for the relaxation of b-CN micelles and of free and bound water were determined according to Equation (6). In our calculations, we used protein aggregation numbers Z (the number of b-CN molecules in a micelle) of 10, 20, and 30. The resulting micelle dipole moments are shown in Figure 8a.

Given the aggregation number Z = 28 obtained by Thurn [20], our maximum estimate of the b-CN micelle dipole moment (7000–9000 D) considerably exceeds the known values of this parameter for folded rigid proteins [73,74]. The permanent dipole moments of globular proteins are often found in the range of 250–550 Debye (D) [75]. The high conformational mobility of an intrinsically disordered protein (IDP) results in the formation of a large fluctuating dipole moment [76]. Disordered b-CN, consisting of long, flexible macromolecules with the charges concentrated at the polar N-termini [77], can produce a large fluctuating dipole moment in the micellar state [30]. The relative spatial orientation of b-CN electric charges in micelles (Figure 1) can enhance the correlations among interacting electric charges, contributing to the formation of the fluctuating dipole moment of the micelle [78]. We believe that this is the first experimentally detected relaxation process associated with a fluctuating dipole moment in a disordered protein. Another argument for assigning this process to the relaxation of a fluctuating dipole moment is its sharp decrease with increasing temperature. Such substantial changes are difficult to reconcile with a permanent dipole moment but can be readily explained by increasing structural fluctuations and reduced correlations among fluctuating charge interactions in an IDP. The activation energy of this process, 30 kJ·mol^−1^ (Figure 7b), significantly exceeds the activation energy for the relaxation of free water as the solvent, and if the process involved the tumbling of a rigid protein with a constant dipole moment, we would expect a value close to the activation energy of solvent relaxation. That is, the measured activation energy of protein relaxation does not contradict the proposed model of a fluctuating dipole moment in b-CN. Regarding possible contributions from relaxation mechanisms associated with the accumulation of ions near interfaces, the system under study contains relatively few free ions (water was used as a solvent), and such processes are generally observed at lower frequencies [79].

The broad δ-dispersion, centered at 10^8^–10^9^ Hz (Figure 6), is the signature of bound, or hydration, water. The relaxation dynamics of bound water in a b-CN micellar solution is characterized by a relaxation time on the order of nanoseconds and an activation energy of 27 kJ/mol (Figure 7), nearly twice that of free water, whose relaxation time and activation energy are close to those of pure water (Figure 6 and Figure 7) [65]. The Kirkwood–Fröhlich Equation (3) was applied to calculate the amounts of free and bound water in the studied systems, which are proportional to the dielectric strengths of γ- and δ-dispersion, respectively. The maximum amount of water bound by b-CN micelles was observed at a low temperature (2 °C) and was equal to 1.1 wt.% of the water present. As the temperature increased to 20 °C, the protein hydration level decreased to 0.81 wt.% owing to increased water mobility and reduced water binding to the protein. Water bound to IDPs is known to exhibit more restricted dynamics than water bound to well-structured proteins [31,33,80], owing to constraints on water molecules arising from the greater number of accessible side chains and electric charges in the polar regions of IDPs, which promote ordered water clustering within collapsed disordered domains [81,82]. Thus, the measured activation energy of 27 kJ·mol^−1^ for bound-water relaxation does not contradict these observations.

The obtained experimental data, particularly the results for the Cole–Cole broadening parameter α, helped us estimate the dynamical hierarchy of dipole–dipole correlations in the studied system. To explain the results obtained for water, it is appropriate to apply the long-established “wait and switch” relaxation mechanism, proposed [83] as an alternative to Debye-like relaxation, which describes the reorientation of water dipoles through Brownian rotational motion [41]. The latter model is now considered unlikely because the rigid hydrogen-bond network strongly restricts the free rotation of water molecules [84,85]. According to the current concept of tetrahedral ordering of water molecules in the hydrogen-bond network, dipole moments can be reoriented only with the aid of orientational defects [84,85,86] or through ionic defects formed by the migration of H_3_O^+^ and OH^−^ ions [87]. The stepwise jumps involving the disruption and “re-switching” of hydrogen bonds are now considered an alternative mechanism by which water molecules sequentially change the orientation of their dipole moments. Using this model and experience gained from its application to different aqueous systems [38,39,43,45,46,47,50], we determined *N*_τ_ as a measure of the spatiotemporal correlations in the dielectric process required for the complete relaxation of each component of the studied system (Equation (4)). The algorithm for calculating *N*_τ_ from the experimentally obtained temperature dependences of the Cole–Cole parameter α is presented in the SM.

Briefly, Equation (4) was used to fit the experimental data for α to obtain the number of relaxation events required for complete relaxation of the system, *N*_τ_, the experimental relaxation time of the studied dipole ensemble, τ, and the duration of a single dipole reorientation event $\tau_{0}$. The obtained results for *N*_τ_ are shown in Figure 8b. It is clear that these characteristics are formal parameters of the model used to represent the studied processes. Nevertheless, the comparison of the three detected relaxation processes, the temperature dependence of *N*_τ_ values, and the relaxation times (Figure 7b) allows us to draw certain conclusions about the molecular details of the experimentally observed relaxation processes. As noted at the beginning of this section, the parameter *N_τ_* provides a model-dependent measure of the number of elementary reorientation events required for complete relaxation of the examined dipole fraction. Moreover, the greater the deviation of the experimental parameter α from 0, the slower the relaxation process, since for complete relaxation it is necessary to overcome stronger constraints imposed by the molecular environment. Figure 8b shows that the relaxation of both free and bound water is comparatively facile: at room temperature, the value of *N_τ_* is close to 1, or in other words, the relaxation of both water fractions is close to the Debye type, i.e., the process of dipole reorientation occurs practically without perturbing neighboring molecules. This is entirely consistent with the “wait and switch” relaxation model, in which a water molecule simply waits for a structural or ionic defect to enable dipole reorientation. However, a decrease in temperature slightly hinders this process, increasing the *N*_τ_ value, apparently because of stronger hydrogen bonds. Nevertheless, although the peak-broadening results indicate similar relaxation mechanisms for both water fractions, apparently because of the associative structure of water, the dynamics of their relaxation differs dramatically. Figure 7b shows that bound-water relaxation is two orders of magnitude slower than free-water relaxation and has a higher activation energy. In our opinion, the most reasonable explanation for this fact lies in the spatial constraints on bound-water reorientation and the influence of the protein surface, which carries numerous electrical charges. Figure 8b shows that the process of dielectric relaxation of micellized b-CN is more complex, and the *N_τ_* value in the 0–25 °C temperature range is about 5, which indicates the cascade nature of b-CN relaxation, in which long-range structural correlations among protein chains bearing numerous electrical charges are clearly involved. This conclusion is supported by the behavior of *N_τ_* with increasing temperature. Clearly, increased intra- and intermolecular mobility of the protein disrupts the long-range correlations of dipole–dipole and dipole–ion interactions, and the relaxation process involves fewer elementary local dipole reorientation events.

## 3. Materials and Methods

### 3.1. Chemicals

To prepare the b-CN micellar solutions, we used a protein with a molecular weight of approximately 24 kDa, extracted from bovine milk by rennet coagulation and purchased from Lactalis (Laval, France). Dielectric measurements were performed using 10 mg/mL aqueous b-CN solutions. To prepare a stock solution of b-CN (20 mg/mL), the required amount of protein was weighed out, and the necessary volume of ultrapure water with a specific conductivity of 18.2 Mohm*cm, produced with a HyperPureX EUS 13 system (Hyperpurex Instrument Technology, Shanghai, China), was added. The resulting suspension was incubated for 12 h at 4 °C and then filtered through a syringe filter (0.22 μm, hydrophilic PVDF, Winstar). The exact protein concentration in the stock solution after filtration was determined spectrophotometrically using the extinction coefficient $\varepsilon_{280}$ (0.1%) = 0.486 [88]. The pH of the prepared solution was 7.45.

### 3.2. DLS Experiments

To confirm the micellar state of the studied b-CN system, dynamic light scattering (DLS) was used. The DLS experiments were performed using a Photocor Compact-Z particle-size analyzer (Moscow, Russia) at a scattering angle of 90°. The hydrodynamic radii of b-CN particles were measured in solutions with protein concentrations of 1.0 and 13.4 mg/mL over a temperature range of 5–25 °C.

### 3.3. Dielectric Measurements

Dielectric measurements were conducted over a frequency range from 1 Hz to 67 GHz in three sequential stages. In the first stage, the dielectric spectra of b-CN solutions were recorded in the frequency range of 1 Hz–10 MHz using the Alpha frequency-response analyzer included in the Novocontrol BDS-80 measurement system. Data acquisition and processing were performed using licensed WinDeta software (version 2.0). A parallel-plate capacitor with 12 mm-diameter electrodes was used as the measuring cell; a 0.5 mm-thick fluoroplastic spacer determined the distance between the electrodes. The cell was calibrated using air and benzene at 20 °C.

In the second stage, measurements were performed in the 1 MHz–1 GHz range using an E4991A radio-frequency analyzer, which was also part of the Novocontrol BDS-80 system. The samples were placed in a similar parallel-plate capacitor.

In the third stage, dielectric spectra were recorded in the 100 MHz–67 GHz range using an Agilent N5247A PNA-X network analyzer. Data were recorded using integrated, licensed Agilent 85070 software. A 10 mm-diameter coaxial probe calibrated with Milli-Q deionized water at 20 °C served as the measuring cell.

The three frequency segments were joined by adjusting the edge capacitance of the measuring cells so that the values of the water dielectric constant agreed within ±0.5% at the midpoint of each overlapping frequency range.

**Figure 9:**
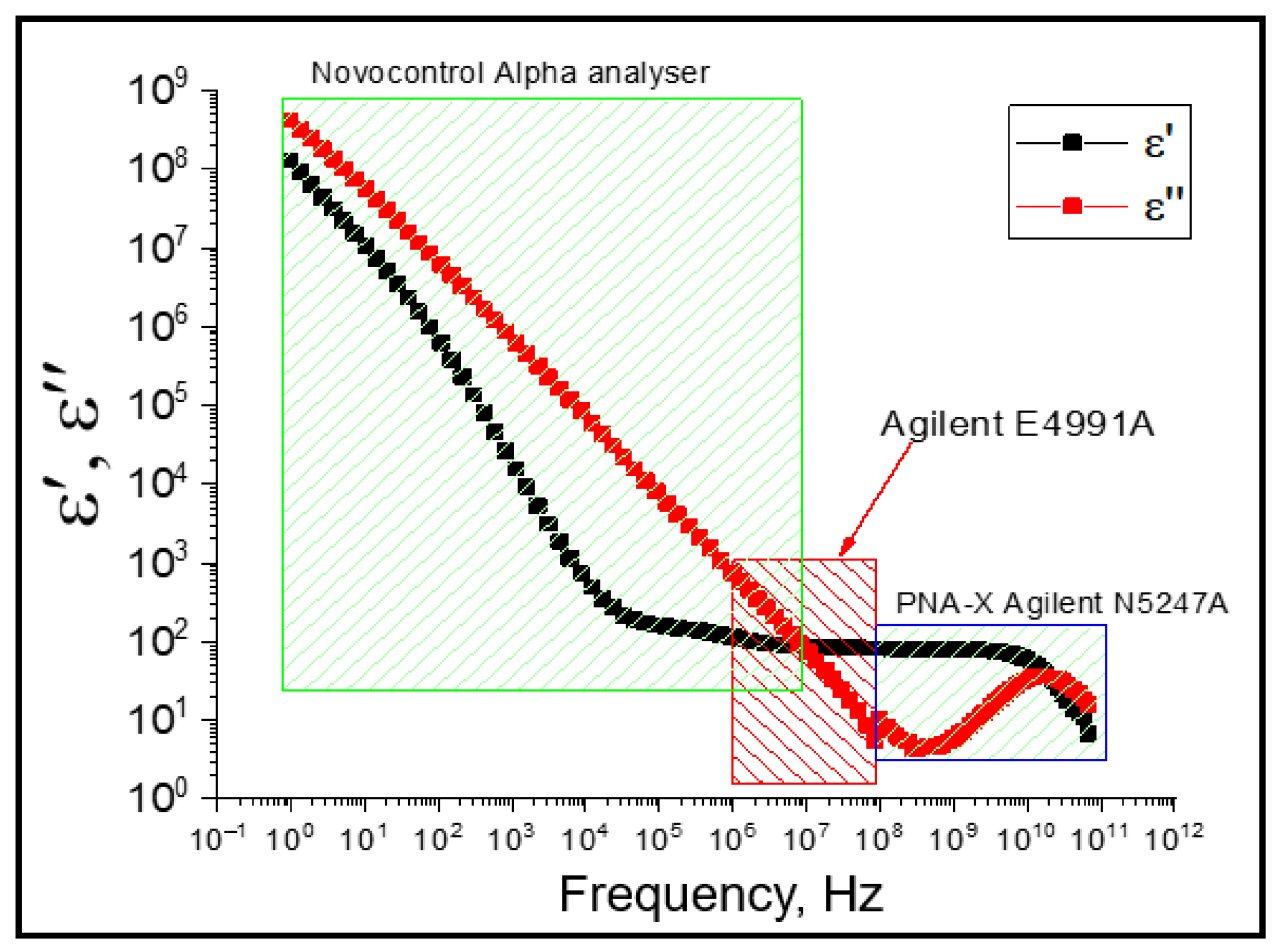
Frequency range of the dielectric experiment covered by the three measurement devices. Data are shown for a 10 mg/mL b-CN solution at 20 °C.

Dielectric measurements were carried out over the temperature range from 0 to 25 °C in 2 °C increments. Temperature control and stabilization were provided by the Quatro system during the first two stages and by the LOIP LT 900 thermal stabilizer during the third stage. The spectra obtained across the three stages were combined to form a broadband dielectric spectrum over the frequency range of 1 Hz to 67 GHz (Figure 9).

To approximate the dielectric relaxation spectra and determine relaxation parameters, we used a superposition of three phenomenological Cole–Cole (CC) functions, the low-frequency Jonscher term, and the direct current (DC) conductivity term, as described by Equation (6). The Jonscher term accounts for the cumulative residual influence of low-frequency processes outside the measurement window, such as electrode polarization. The parameters ε_s_ and ε_∞_ were obtained as the extrapolated limits of permittivity at low and high frequencies, respectively. Dielectric relaxation parameters were calculated using the Datama software package (version 2.0) [89].

## 4. Conclusions

For many decades, protein function was associated with a rigid three-dimensional structure. However, in recent years, interest has grown rapidly in intrinsically disordered proteins that successfully perform diverse functions in living systems and various biotechnological applications. Unfortunately, to date, insufficient structural information has been collected on this novel protein family to analyze its structure–function correlations.

To investigate the dynamical structure of the intrinsically disordered protein b-CN in an aqueous solution, broadband dielectric spectroscopy was used. Dielectric measurements conducted over a frequency range spanning more than ten orders of magnitude, from 1 Hz to 67 GHz, revealed three Cole–Cole relaxation processes assigned to b-CN in the micellar state and to bound and free water. The lowest-frequency process, which has an extremely high amplitude and is centered at 10^6^–10^7^ Hz, was assigned to the relaxation of the disordered protein, caused by the formation of a large fluctuating dipole moment in flexible macromolecules with a complex dynamic charge distribution assembled into well-organized micellar aggregates. The dielectric relaxation of micellized disordered b-CN is rather complex, indicating the cascade nature of protein relaxation, which involves long-range structural correlations among protein chains containing numerous electrical charges. The other two relaxation processes were identified as the relaxation of free water (20–30 GHz) and bound (hydration) water (centered at 10^8^–10^9^ Hz). The obtained experimental data, particularly the results for the Cole–Cole broadening parameter, were used to estimate the dynamical hierarchy of dipole–dipole correlations required for the relaxation of each component of the studied system. The results show that the relaxation of free and bound water is close to Debye-type behavior, i.e., dipole reorientation occurs with little perturbation of neighboring water molecules. This is entirely consistent with the “wait and switch” model of dielectric relaxation, in which a water dipole simply waits for the arrival of a structural or ionic defect before reorientation. Despite the similarity of the relaxation mechanisms of the two water fractions, bound-water relaxation is two orders of magnitude slower than free-water relaxation. In our opinion, the most reasonable explanation for this difference lies in the spatial constraints on bound-water reorientation imposed by the protein surface, which carries a large number of electric charges.

To the best of our knowledge, this is the first experimental study on the dielectric properties of the intrinsically disordered protein b-CN, together with the relaxation behavior of free and bound water associated with the protein. The results provide new information on the dynamical structure of the disordered protein, offering unique insights into dynamic processes that occur on different scales and arise from the protein’s disordered structural organization.

## Data Availability

The data in this study are available on reasonable request from the corresponding author.

## Supplementary Materials

The following supporting information can be downloaded at: https://www.mdpi.com/article/10.3390/ijms27198810/s1.
