# Supplementary material for: Dielectric Response of Micelles Built from Intrinsically Disordered Beta-Casein
Source: Int J Mol Sci. 2026 Oct 1;27(19):8810. doi: 10.3390/ijms27198810 (PMC13665254; doi:10.3390/ijms27198810)
Supplement: Supplementary file 1 [file ijms-27-08810-s001.zip › ijms-4508769-supplementary.pdf]

Supporting Information

Dielectric response of micelles built from intrinsically disordered beta-casein

Yuriy F. Zuev[1*], Denis A. Smolei[1], Polina V. Skvortsova[1], Liliya R. Bogdanova[1], Artur A. Galiullin[2], Olga S. Zueva[3], Svetlana R. Derkach[4], Ivan V. Lunev[2]

[1] Kazan Institute of Biochemistry and Biophysics, FRC Kazan Scientific Center, Russian Academy of Sciences, Kazan 420111, Russian Federation; yufzuev@mail.ru (Y.F.Z.); denissmolej@gmail.com (D.A.S.); skvpolina@gmail.com (P.V.S.); chemli@mail.ru (L.R.B.)

[2] Institute of Physics, Kazan (Volga Region) Federal University, Kazan 420021, Russian Federation; lounev75@mail.ru (I.V.L.); www.ag95@mail.ru (A.A.G.)

[3] Institute of Electric Power Engineering and Electronics, Kazan State Power Engineering University, Kazan 420066, Russian Federation; ostefzueva@mail.ru (O.S.Z.)

[4] Institute of Natural Sciences and Technology, Murmansk Arctic University, Sportivnaya Str. 13, 183010 Murmansk, Russian Federation; derkachsr@mauniver.ru (S.R.D.)

* Correspondence: yufzuev@mail.ru (Y.F.Z.)

1. Calculating of the reorientation events number N_τ, required for complete relaxation

The spatio-temporal correlation coefficient N_τ was calculated using the dependence α = f (ln τ) (Eq. 4), according [1]. The experimental dependences α = f (ln τ) (fig. S1) were minimized using the least-squares method to obtain parameter N_τ, the number of reorientation events required for complete relaxation in the observed relaxation process, or the relative spatiotemporal correlation of dipole-dipole interactions among the relaxing dipoles. The Levenberg-Marquardt method was used for this approximation.

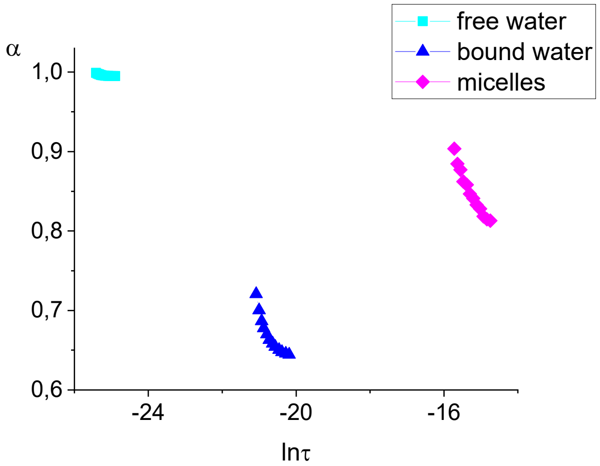

Figure S1. Experimental dependences α = f (ln τ) for three relaxation components.

[1] L. Latypova, A. Puzenko, A. Gural, G. Barshtein, A. Bogdanova, Yu. Feldman, Water state in hemoglobin solutions. Microwave dielectric spectroscopy study, IEEE Trans. Diel. Electr. Ins. 30 (2023) 1649-1656 https://doi.org/10.1109/TDEI.2023.3283465.

2. Calculation of the average dipole moment of b-CN micelles

Calculations were performed in two stages. First, we have calculated the Fröhlich function using the measured dielectric spectra:

$$B(T) = \Delta\varepsilon(T)\frac{2\varepsilon_s(T)+\varepsilon_\infty}{3\varepsilon_s(T)} \quad \text{S1}$$

After calculating the Fröhlich function, the dipole moment of micelles was calculated:

$$\langle\vec{M}\rangle = \sqrt{3\varepsilon_0 k_B V_0 B(T)}\,. \quad \text{S2}$$

3. Comparison of the dielectric permittivity spectra for 10 mg/ml b-CN solution at three different temperatures.

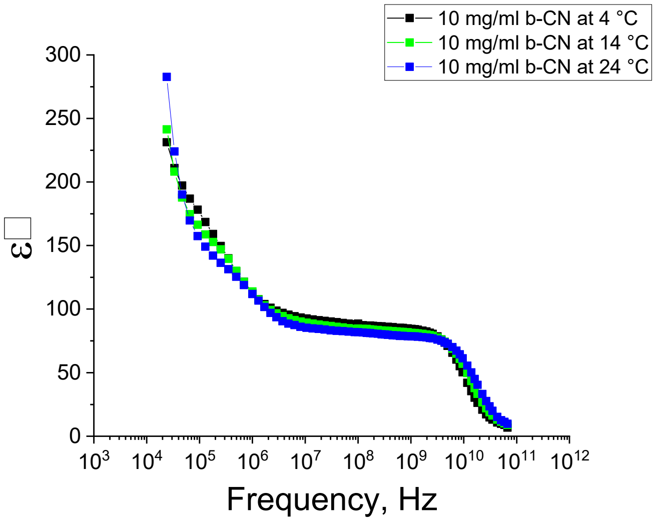

Figure S2. The typical example of obtained experimental data - dielectric permittivity (ε′) spectra for 10 mg/ml b-CN solution at 4, 14 and 24°C.
